# Holographic acoustic elements for manipulation of levitated objects

**DOI:** 10.1038/ncomms9661

**Published:** 2015-10-27

**Authors:** Asier Marzo, Sue Ann Seah, Bruce W. Drinkwater, Deepak Ranjan Sahoo, Benjamin Long, Sriram Subramanian

**Affiliations:** 1 Deparment of Mathematics and Computer Engineering, Public University of Navarre, Campus Arrosadia, 31006 Pamplona, Spain; 2 Department of Computer Science, University of Bristol, Woodland Road, Bristol BS8 1UB, UK; 3 Ultrahaptics Ltd, Engine Shed, Station Approach, Bristol BS1 6QH, UK; 4 Department of Mechanical Engineering, University of Bristol, University Walk, Bristol BS8 1TR, UK; 5 Department of Informatics, University of Sussex, Falmer, Brighton BN1 9RH, UK

## Abstract

Sound can levitate objects of different sizes and materials through air, water and tissue. This allows us to manipulate cells, liquids, compounds or living things without touching or contaminating them. However, acoustic levitation has required the targets to be enclosed with acoustic elements or had limited manoeuvrability. Here we optimize the phases used to drive an ultrasonic phased array and show that acoustic levitation can be employed to translate, rotate and manipulate particles using even a single-sided emitter. Furthermore, we introduce the holographic acoustic elements framework that permits the rapid generation of traps and provides a bridge between optical and acoustical trapping. Acoustic structures shaped as tweezers, twisters or bottles emerge as the optimum mechanisms for tractor beams or containerless transportation. Single-beam levitation could manipulate particles inside our body for applications in targeted drug delivery or acoustically controlled micro-machines that do not interfere with magnetic resonance imaging.

## Abstract (web summary)

Acoustic waves can be used to levitate objects in air, water and tissue. Here, the authors introduce a single-beam scheme to facilitate the translation, rotation and manipulation of levitated objects in 3D, and design acoustic structures such as tweezers, twisters and bottle traps.

## Graphical abstract

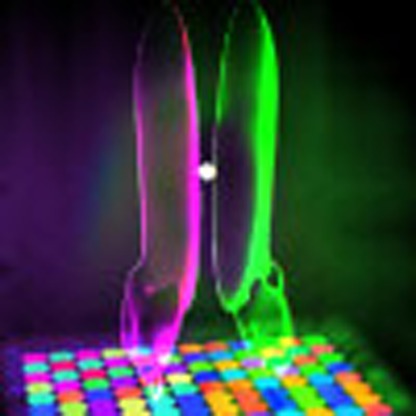

Acoustic waves can exert radiation forces^1^ and form acoustic traps at points where these forces converge permitting the levitation of particles of a wide range of materials and sizes^2^ through air^3^, water^4^ or biological tissues^5^. This is of paramount importance for crystallography^6^, cell manipulation^7^, lab-on-a-chip scenarios^8^, biomaterials^9^, containerless transportation^3^,^10^ and even the levitation of living things^11^.

With previous acoustic levitators, the trapped particles had to be enclosed by acoustic elements^3^,^10^,^12^,^13^,^14^. Single-sided (or single-beam) levitators only exerted lateral trapping forces^15^,^16^, pulling forces^17^,^18^ or required the use of an acoustic lens^19^. Furthermore, translation^3^,^12^,^13^,^14^ and rotation^20^ of the traps were limited.

Single-axis levitators^3^,^6^,^10^,^13^ are a common arrangement for generating acoustic traps. They consist of an acoustic transducer and a reflector or another transducer above it. This generates a standing wave between the two elements and the nodes of the wave act as trap. By changing the phase difference between the transducers, the traps move in a single dimension without mechanical actuation. Various configurations for two-dimensional manipulation have been explored, for example, a flat array of transducers and a parallel reflector provides movement within the plane of the array^3^,^13^. Alternatively, an inward-facing circular array of transducers can translate^4^,^12^ and rotate^20^ a particle within the circle. Three-dimensional (3D) translation is possible with four arrays placed forming a square^14^ and recently with two opposed arrays^21^.

Recent progress has seen custom-made piezoelectric elements being used to create traps with a single-sided device (acoustic tweezers)^15^,^16^. However, these traps only exert lateral forces and thus the particles have to rest on a surface. Pulling forces acting counter to the propagation direction (tractor beams) have been measured in water using triangular-shaped particles^18^ and in air using acoustic bottle beams^17^. Full 3D trapping with a single-sided device has been shown theoretically^22^,^23^ and a static underwater 3D trap has recently been reported^19^. Nonetheless, a physical acoustic lens was required, introducing considerable energy loss^16^ and fixing the position of the trap to the focal point.

Controlled 3D trapping, translation and rotation with a single-sided array would enable acoustic tweezers to become the larger-scale counterparts of optical tweezers^24^, opening up applications in materials processing, micro-scale manufacturing and biomedicine.

Here we demonstrate simultaneous 3D acoustic trapping, translation and rotation of levitated particles using a single-sided array operating in air. This is achieved by optimally adjusting the phase delays used to drive an array of transducers; in this way unprecedented acoustic structures are generated without resorting to physical lenses, custom transducers or mechanical actuation. Our approach generates optimum traps at the target positions with any spatial arrangement of transducers and significantly enhances previous manipulators^3^,^12^,^14^. We report three optimum acoustic traps: tweezer-like twin traps, a novel acoustic phenomenon with the ability to also rotate objects; twister-like vortex traps, whose levitation capabilities were shown theoretically^22^,^23^ and recently observed experimentally using a fixed acoustic lens^19^; and bottle-shaped traps, never proven or suggested to levitate objects before^17^. We also introduce the holographic acoustic element framework based on interpreting the phase delays as a holographic plate that combines the encoding of identifiable acoustic elements. The framework permits the analysis and efficient generation of acoustic traps as well as comparisons with optical traps. This work brings the advantages of optical tweezing (that is, single-beam, rotation, holographic control and multiple particles)^24^ to the efficiency and versatility of acoustic levitation and could lead to the development of powerful tractor beams, 3D physical displays or acoustically controlled *in vivo* micro-machines that do not interfere with magnetic resonance imaging.

## Results

### Universal optimizer

We characterize a 3D trap as a point towards which the forces converge from all directions. More explicitly, the Gor’kov potential^1^ defines a field, the gradient of which gives the forces exerted on small spheres; therefore, the Laplacian operator applied to the Gor’kov potential represents the trapping strength at a certain point. The Gor’kov Laplacian function at one position in space can be expressed as a nonlinear infinitely differentiable function with the phase delays (modulations) applied to the transducers as the only variables. With this function and the gradient of its variables, we employ a Broyden–Fletcher–Goldfarb–Shanno (BFGS) optimizer^25^ to obtain the phase modulations for the transducers so that when driven with a reference signal the generated acoustic field exerts maximum trapping forces on a particle situated at the target point. Our formulations of the Gor’kov Laplacian and its gradient enable real-time optimization.

Maximizing the Gor’kov Laplacian at a point sets the phase modulation of the transducers to generate a focal point at that position. In theory, a focal point can trap a particle exactly at its centre, where all the amplitude gradient forces cancel each other and the velocity gradient forces drag the particle in; amplitude gradients push the particles from high-amplitude regions to low-amplitude ones, whereas velocity gradients displace particles towards regions with high complex gradients of the acoustic field (see Methods, equation (3)). However, a focal point is only a theoretical solution; experimentally, dense particles are repelled by the focal point^22^and it is not possible to levitate particles around a focal point in a stable manner (Supplementary Fig. 1 and Supplementary Movie 1). Consequently, our optimizer uses an objective function that simultaneously maximizes the Gor’kov Laplacian and minimizes the pressure amplitude at the target point. These silent acoustic traps are the counterpart of dark optical traps^26^. In addition, weights are applied to each component of the Gor’kov Laplacian to control the trapping strength in each dimension (see Methods, equation (9)).

**Figure 1:**
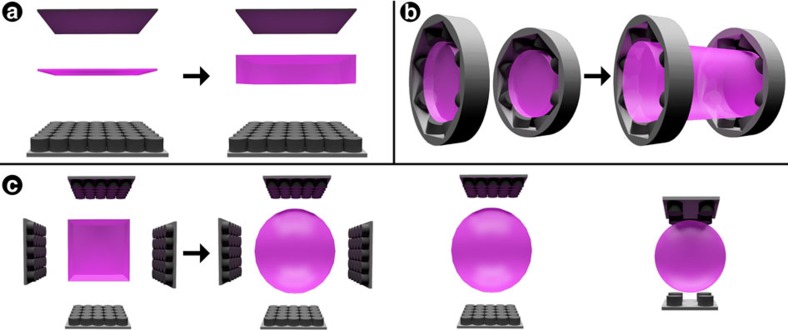
Schematic rendering of the working volume of previously suggested manipulators. The magenta volume represents the area within which the particles can be translated in a controlled manner. To the left of the arrow the previous working volume is shown and to the right the working volumes using our approach. (**a**) With our method an acoustic reflector on top and transducers on the bottom can move objects in 3D, previously it was only possible in the *z* plane^3^,^13^. (**b**) Ring-shaped arrangements can now translate particles inside the tube formed when various rings are placed together, before it was only possible inside a single ring^4^,^12^. (**c**) Ochiai *et al.*^14^ four-array manipulator expands its working volume, can work with only two arrays and a low density of transducers.

**Figure 2:**
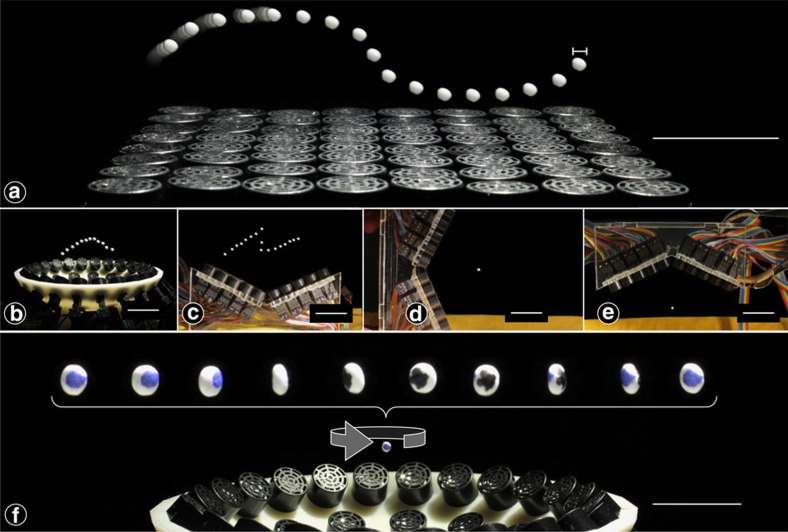
Pictures of one-sided levitation in mid-air. Expanded polystyrene particles ranging from 0.6 to 3.1 mm diameter are levitated above single-sided arrays. The acoustic transducers (10 mm diameter) are driven at 16 Vpp and 40 kHz. (**a**–**c**) The particles can be translated along 3D paths at up to 25 cm s^−1^ using different arrangements and without moving the array. (**c**–**e**) The traps are strong enough to hold the spheres and counteract gravity from any direction. (**f**) Asymmetric objects, such as ellipsoidal particles, can be controllably rotated at up to 128 r.p.m. Scale bars represent 2 mm for the particle in **a** and 20 mm for the rest.

This optimization method can be applied to scenarios with reflectors and any spatial arrangement of acoustic elements. Therefore, we can use it to control and enhance previously suggested manipulators. The improvements on the working volume for some arrangements from the literature^3^,^12^,^14^ are presented in Fig. 1 as a comparative qualitative representation (Supplementary Movie 2). This illustrates the benefit of using an optimization approach over the current positioning algorithms. More importantly, we show here that the optimization method can for the first time trap, translate and rotate particles using single-sided arrays (Fig. 2). Depending on the spatial arrangement of the array and the weights selected for each dimension, different acoustic traps are created. For a detailed description of the arrangements, see Supplementary Figs 2 and 3 and Supplementary Note 1.

### Optimal single-beam acoustic traps

The three optimal traps that emerge as optimum solutions for single-sided arrays are twin (Fig. 3), vortex (Fig. 4) and bottle (Fig. 5) traps. Experimental measures of these traps are presented in Supplementary Figs 4–8 and Supplementary Notes 2 and 3.

Acoustic traps can be analysed in terms of the origin of the exerted forces; namely, radiation forces are generated by amplitude gradients or velocity gradients^1^ (see Methods, equation (3)). In addition, phase singularities can be used to characterize the traps. Phase singularities are regions with zero amplitude and thus where the phase is not defined^27^.

**Figure 6:**
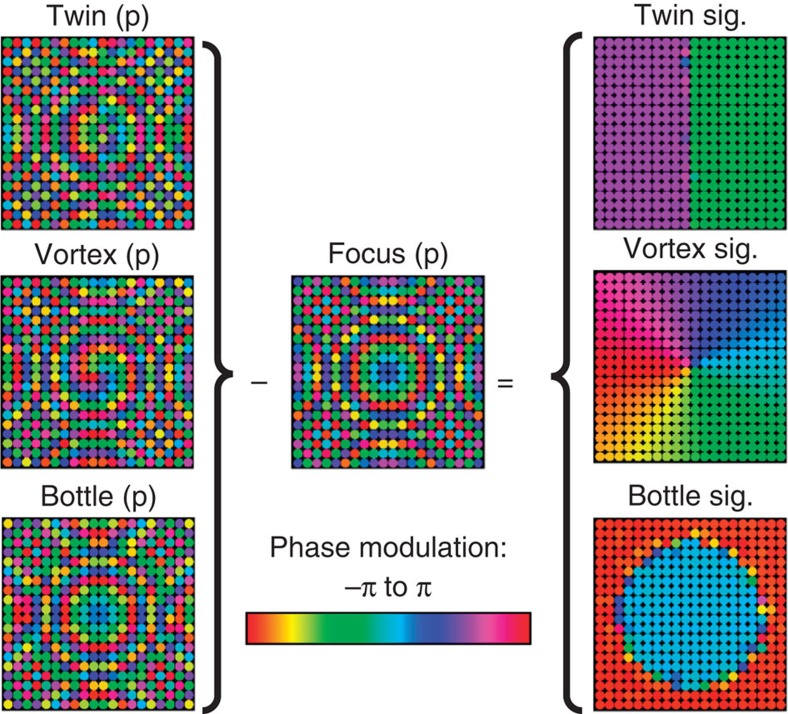
Holographic signatures of the three optimal traps. Phase modulations of the transducers for generating each of the traps (left), their decomposition into a focusing element (centre) and the holographic signatures (sig., right).

As a novel method to analyse traps, we introduce the concept of holographic acoustic elements. The phase modulation applied to the transducers is interpreted as a holographic plate that when driven with a reference signal renders an acoustic field. In our case, the traps are encoded as the combination of two holographic acoustic elements: a holographic acoustic lens that generates a focal point at the trap position and an extra element dependent on the type of trap (Fig. 6). The lens is obtained by making all the emitted waves coincide in phase at the focal point. By subtracting this lens from the optimized total plate, the holographic signature of the trap is obtained. The signature is an interesting feature for analysing the traps as to some extent it is invariant to the levitation position and can be compared with existing holographical optical traps^24^,^26^.

Twin traps emerge when equal weights are specified in v-shape arrangements or a large *x* axis weight is used for other arrangements. These traps have two finger-like cylindrical regions of high amplitude, which tweeze the particle with amplitude gradients in the *x* direction. Velocity gradients constrain in the other two axes. A plane phase singularity (that is, two-dimensional) occurs between the cylinders in the *x* plane. The holographic signature has a π-phase difference between the two halves of the array. By rotation of the reference co-ordinate system or the holographic signature, the tweezer structure and the clamped particle can be rotated. Twin traps are shown in operation in Fig. 2 and have never been reported theoretically or experimentally in acoustics or optics.

Vortex traps emerge when equal weights are used in a hemispherical cap or a flat array. The *xy* section of the trap shows a high-amplitude ring that generates lateral trapping forces with amplitude gradients. Along the *z* axis, the trapping force is due to velocity gradients and the phase is a 3D corkscrew spiralling around a line phase singularity (that is, one-dimensional). The holographic signature is a helicoidal pattern. A particle trapped in this vortex trap spins around its own axis following the signature direction due to transfer of angular momentum^28^,^29^. In our experiments, only small particles could be trapped (diameter <0.12*λ*=1 mm), see Supplementary Fig. 9, Supplementary Note 4 and Supplementary Movie 3 for further details. Vortex traps in acoustics have been shown theoretically^22^,^23^ and recently experimentally using a fixed acoustic lens^19^. We note that the acoustic vortex trap that emerges from our optimizer is equivalent to an optical vortex^26^ (Supplementary Note 5 and Supplementary Fig. 10).

Bottle traps emerge in all the arrangements when large weights are applied to the direction of propagation (*z* axis). These traps create a high-amplitude cage around the levitation point and all the forces result from amplitude gradients. A point phase singularity (that is, zero-dimensional) is found at the trap centre. Here the holographic signature is a circular region of π-phase difference. Bottle traps have been reported in acoustics^17^ but their ability to levitate particles was never suggested or proved; in optics, they have been generated by intersecting two laser beams with different modes^30^.

Twin traps and vortex traps have a similar working volume for the same arrangements (Supplementary Fig. 11 and Supplementary Note 6) and this is comparable to the working volume of a standard single-axis levitator. The *z* axis range with the tested single-sided arrays was up to 40 mm. This range is sufficient for many applications and could be increased by using more powerful transducers, a different host medium or if it not were necessary to defy gravity (for example, underwater applications). Bottle traps were limited in working volume since the lateral forces were weak, and the bottle shape was not maintained when the trap was generated off-centre.

Particles could be transported horizontally at up to 26 cm s^−1^ (Supplementary Tables 1 and 2 and Supplementary Note 7), this speed being limited by the update rate of our custom electronics (Supplementary Note 1). Vortex and twin traps achieved similar horizontal transport speeds and much higher vertical speeds than a traditional standing wave created with a two-sided device. Twin traps are not symmetric around the *z* axis leading to faster transport speeds in the *y* direction than in the *x* direction. Bottle traps were limited in transport speed because of their relatively weak lateral forces. Other systems have reported maintained speeds of up to 4.9 (ref. 3), 3.2 (ref. 23) and 7 cm s^−1^ (ref. 20). The accuracy of particle repositioning (Supplementary Tables 3 and 4 and Supplementary Note 8) was at least 0.4 mm (*λ*/21) and up to 0.05 mm (*λ*/171) depending on the trap and axis, which is comparable to that achieved in previous devices^12^.

Forces of the order of μN were generated on particles due to the trapping forces, this is comparable to previous levitation systems^12^,^17^. The trapping forces are presented as spring constants in Supplementary Fig. 12, Supplementary Tables 5 and 6, and Supplementary Note 9. Twin and vortex traps had lateral forces comparable to a standing wave generated with a traditional single-axis device but the *z*-direction forces were around 30 times weaker. Bottle traps had *z*-direction forces seven times stronger than twin and vortex traps, potentially leading to a greater *z* range, but their low lateral forces diminished their manoeuvrability. That is, it was possible to levitate particles at relatively large distances form the array, but not to reach those positions moving the particle from the central position. In general, pressure amplitude gradients were seen to generate much stronger forces than velocity gradients. The strength of the traps affected negatively the speed of transport but this was caused by the update rate of the electronics. Strong trapping forces require smaller step sizes for stable transport (Supplementary Tables 7 and 8 and Supplementary Note 7) and thus faster updates of the phases.

### Holographic acoustic framework

**Figure 7:**
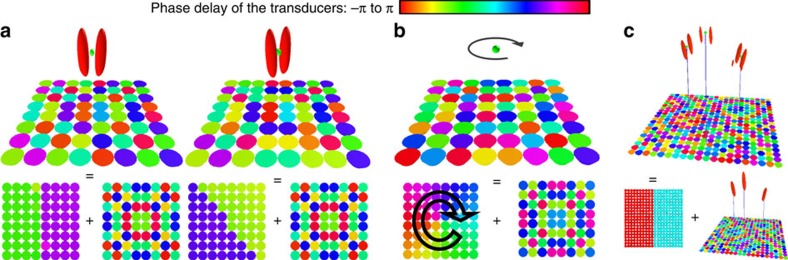
The holographic method permits the creation of traps at different positions by combining a holographic signature with a holographic focusing element. Colour represents the phase modulation of the transducers. The traps are generated at the focal point of the holographic lens. (**a**) Rotating a particle by combining the rotated signature of a twin trap with a focusing lens. (**b**) A vortex trap will transfer orbital angular momentum to the particle with the same direction as the signature. (**c**) Multiple traps can be obtained by adding a signature to the phase modulation that generates focal points at the target locations.

The holographic acoustic element framework can also be used as a fast method to generate traps at different positions, rotate and spin particles as well as to create multiple levitation points (Fig. 7). Any trap can be generated at different locations by adding its signature to the phase delays that generate a focal point at the desired position, thus moving the trap is like refocusing the holographic acoustic lens. Rotating the holographic signature of a twin trap makes the trap structure and the trapped particles to rotate. Vortex traps transfer angular orbital momentum to the levitated particle with the same direction as the signature. And, when a holographic signature is added to a plate that generates multiple focal points, these points get transformed into traps that are of the same type as the added signature.

## Discussion

Until now, only standing waves^3^,^10^,^14^,^20^,^21^ or Bessel beams^4^,^12^ were capable of translating levitated particles. On the other hand, single-sided arrays required an acoustic lens and generated static traps^19^. Here we have presented an optimization method that creates optimal traps at the desired positions with different array geometries. It can directly control previous manipulators offering better results in terms of working volume. More importantly, the method can be applied to single-sided arrays and generates some unprecedented acoustic structures (that is, twin traps).

The introduction of three acoustic structures for the translation and rotation of levitated particles will find applications in tractor beams, containerless handling of matter and tangible displays. Our systems use inexpensive low-power transducers but high-power versions could enable longer range 3D transportation, orientation and assembly of heavier objects. Single-sided devices potentially enable *in vivo* manipulation since the device could be applied directly onto the skin with the manipulation taking place inside the body; similar to an ultrasound scanner but for manipulating particles (that is, drug capsules, kidney stones or micro-surgical instruments). This is a significant advantage over two-sided opposed arrangements, which require the target area to be sandwiched by the arrays; also, single-beam traps do not have repeated patterns that could accidentally trap other particles or generate undesired secondary maxima.

We also introduced the holographic acoustic framework that allows the traps to be generated without iterative methods. A direct link between optical and acoustic trapping has now been established and we expect this to yield further advances in both fields.

## Methods

### Characterizing a Levitation Point

The acoustic radiation force (***F***) exerted on a small spherical particle can be calculated from the gradient of the Gor’kov potential^1^ *U*:




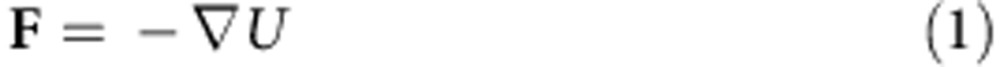




We characterize a levitation point as a maximum of the Laplacian operator (convergence of the gradient) applied to the Gor’kov potential, that is, a point towards which all the forces converge.




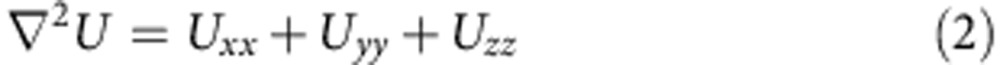




where 
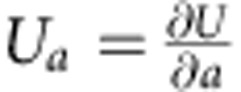
, 
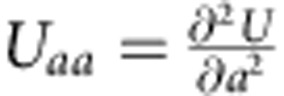
 and *a*=*x*, *y*, *z* are the Cartesian axes.

The Gor’kov potential, *U*, in terms of the complex acoustic pressure (*p*) and its spatial derivatives is given by:




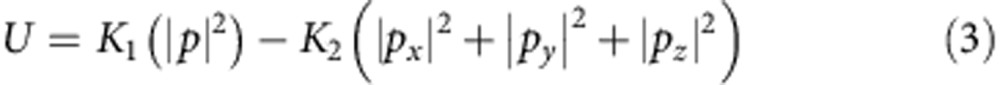







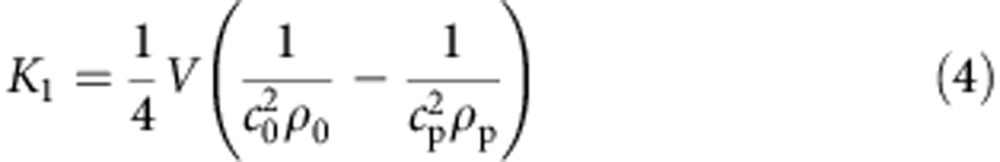







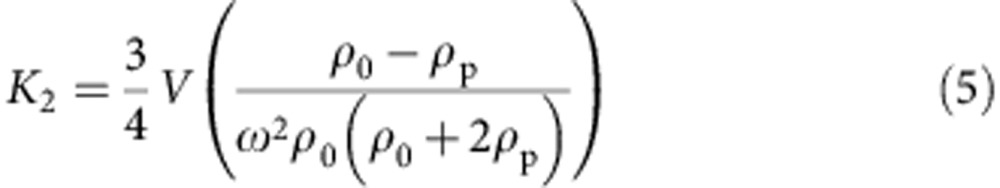




where *V* is the volume of the spherical particle, *ω* is the frequency of the emitted waves, *ρ* is the density and *c* is the speed of sound (with the subscripts 0 and p referring to the host medium and the particle material, respectively). In equation (3), the first term relates to the amplitude gradient and establishes that particles are moved from regions with high amplitude towards regions with low amplitude; the second term relates to the velocity gradient and establishes that particles are dragged into regions with large modulus of the gradient of the complex field.

If an acoustic transducer emits with a constant frequency and amplitude, then the complex pressure that the *j*th transducer creates at a point can be expressed as:




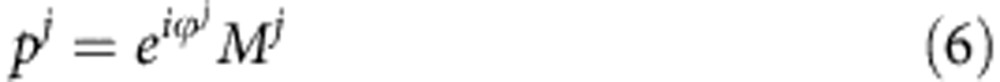




where 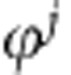 is the phase delay of the transducer and 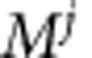 is a complex number that is constant for a given transducer and point in space. Owing to linearity, this also holds true for the spatial derivatives of the pressure, for instance 
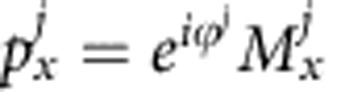
.

To predict the 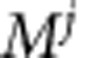 constants and their spatial derivatives, several methods can be used; namely, the matrix method, finite differences or experimental measures. We employ a far-field model of a circular piston source:




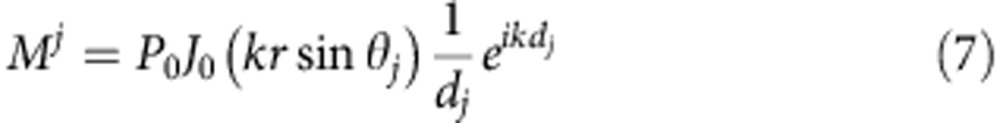




where *P*_0_ is a constant defined by the transducers power, *J*_0_ is a zeroth-order Bessel function of the first kind, *k* is the wavenumber 
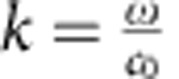
, *r* is the radius of the piston, *d*_*j*_ is the distance between the transducer and the point, and *θ*_*j*_ is the angle between the transducer normal and the point. The piston model was adequate as the simulations matched the experiments for both the complex acoustic field (Supplementary Figs 4–8 and Supplementary Note 3) and the predicted levitation positions (Supplementary Tables 9 and 10 and Supplementary Note 10).

The total acoustic field (*p*) generated by *N* transducers is the addition of the individual fields, that is, 
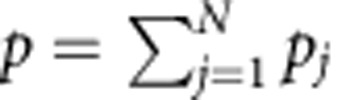
. This also holds true for its spatial derivatives, for instance 
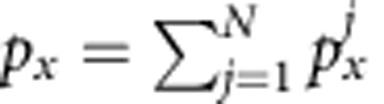
.

Therefore, the Laplacian of the Gor’kov potential can be expressed as a function of the phase delay of the transducers, 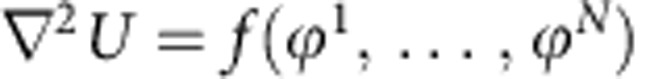.

### Objective function

For a stable levitation trap, the Laplacian of the Gor’kov potential (which we termed the Gor’kov Laplacian) must be maximized and the modulus of the pressure (amplitude) must be minimized. Therefore, the objective function to minimize is:




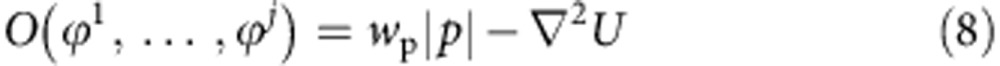




which can be expanded with the addition of individual weights for the Cartesian axes:




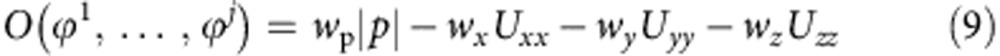




where *w*_*x*_, *w*_*y*_ and *w*_z_ are weights used to accentuate or damp the trapping forces in particular directions; large weights are proportions of 1,000 to 1. *w*_p_ is used to specify the balance between maximizing the Gor’kov Laplacian and minimizing the amplitude; here a value of 1 is used.

### Efficient evaluation of the objective function and its gradient

Each term of the objective function can be explicitly expressed in terms of the pressure and its spatial derivatives:




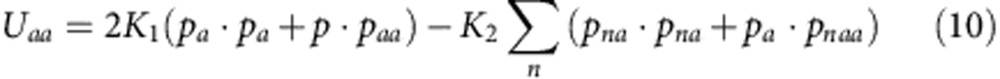







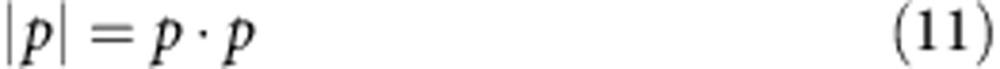




where 
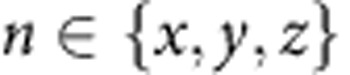
 and the operator ‘·’ is defined as:




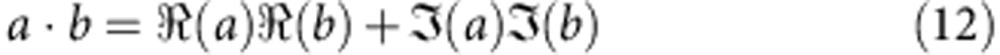




The objective function can be differentiated with respect to the phase of the *j*th transducer by applying the following formula:




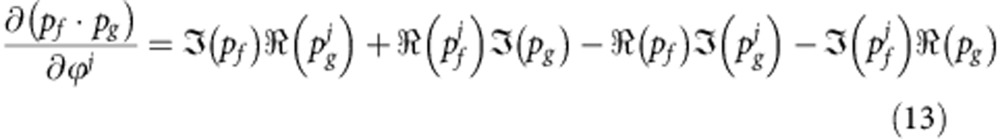




where *f*, *g*∈{, *x*, *y*, *z*, *xy*, *xz*, *yz*, *xx*, *yy*, *zz*, *xxy*, *xxz*, *xyy*, *xzz*, *yzz*, *xxx*, *yyy*, *zzz*}. That is, *p*_*f*_ and *p*_*g*_ can be the complex pressure or any of its spatial derivatives.

Using equations (10)–(13), 21, 23, 24 we can assemble the objective function (equation (9)) and its gradient for a given point in space (that is, the required levitation point). Our algorithm is highly efficient as evaluating the target function (equation (9)) or its gradient at one point has time complexity *O*(*N*) where *N* is the number of transducers. In addition, the employed optimizer (BFGS) presents superlinear convergence.

Once the complex constants 
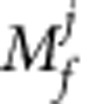
 for the desired levitation point have been calculated, the Gor’kov Laplacian and its gradient can be evaluated as follows: First, calculate the pressure and its spatial derivatives that each transducer creates at the target point given the transducer phase delay, 
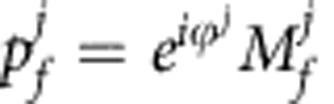
. Second, calculate the total pressure and its spatial derivatives, 
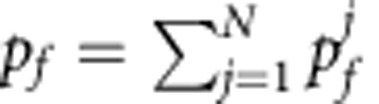
. Third, evaluate the objective function (equation (9)) using equations (10)–(12), 21, 23 and the previously calculated *p*_*f*_. And finally, calculate the gradient of the objective function by taking the derivative of the objective function over each of its variables 
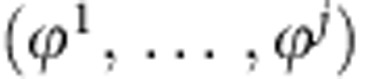
. This can be done by applying equation (13) to each term of equations (10) and (11) using the previously calculated *p*_*f*_ and per transducer 
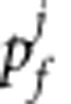
.

### BFGS optimization

The BFGS algorithm is an iterative method for unconstrained nonlinear optimization. Contrary to Newton’s method, it does not require the time-consuming calculation of the inverse Hessian matrix. At every step, the optimizer needs to evaluate the function to minimize and its gradient.

For the linear search strategy, we employed the Armijo–Camino rule with *α*=1, *β*=0.5 and *σ*=0.0001. We also tried Basin Hop (temperature=0.1; step size=120) to ensure that the global minimum was found but it was not necessary. In our experiments with 400 transducers, ∼9,000 iterations were sufficient to converge on a solution starting from a random set of phase delays.

BFGS optimizers^25^,^31^ with Basin Hop^32^ have been successfully used before to solve the structure of condensed matter^33^, proteins^34^,^35^ or atoms configurations^32^,^36^. Now, this powerful and versatile approach also brings marked improvements to acoustic levitation.

## 

## Additional information

**How to cite this article:** Marzo, A. *et al.* Holographic acoustic elements for manipulation of levitated objects. *Nat. Commun.* 6:8661 doi: 10.1038/ncomms9661 (2015).

## Supplementary Material

Supplementary Information Supplementary Figures 1-12, Supplementary Tables 1-10 and Supplementary Notes 1-10.

Supplementary Movie 1 2_LevitationFocal. A particle levitating unstably around the focal point until it drops. The particle levitates 19cm. above a flat array. ·3_SpinningOutOfVortex. A particle levitates in a Vortex trap, starts to orbit and then gets ejected.

Supplementary Movie 2 1_MainVideo. Main video that summarizes the paper. From 0:09 to 0:36, single-sided levitation, rotation and spinning of particles are shown simultaneously in the simulation and experiments. From 0:39 to 0:47, full acoustic trapping with levitators placed at different angles. From 0:49 to 1:54, explanation of the optimization method. Amplitude iso-surface, amplitude slices, phase slices, potential and holographic decomposition of Twin traps (2:01 to 2:21), Vortex traps (2:23 to 2:45) and Bottle traps (2:45 to 3:03). From 3:05 to 3:29, dynamic holographic decomposition of moving traps. From 3:30 to 3:49, holographic method for multiple traps. From 3:50 to 4:10, enhancing previous manipulators.

Supplementary Movie 3 3_SpinningOutOfVortex. A particle levitates in a Vortex trap, starts to orbit and then gets ejected.

## Figures and Tables

**Figure 3:**
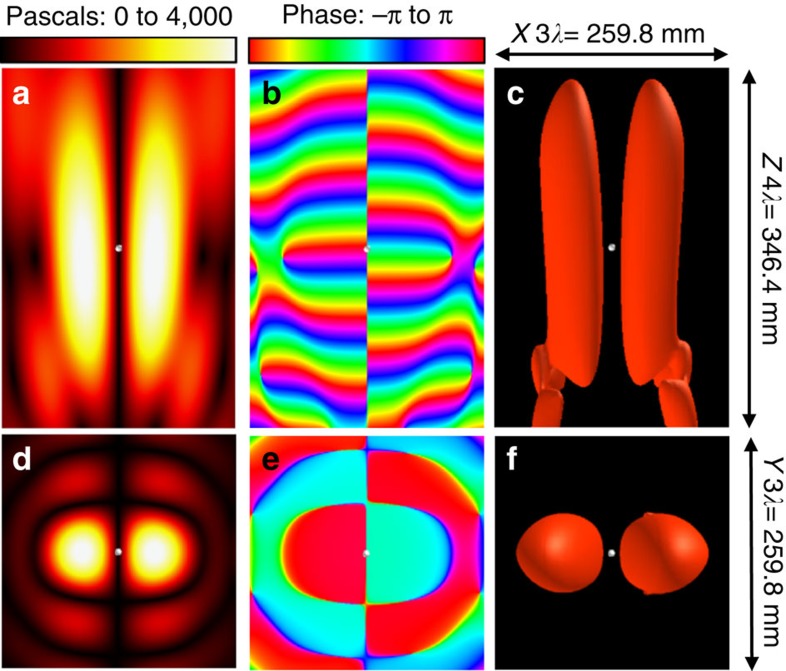
Twin trap generated with a flat 20 × 20 array 12 cm above the centre. Amplitude field (**a**,**d**), phase field (**b**,**e**) and amplitude isosurfaces of 2 kPa (**c**,**f**).

**Figure 4:**
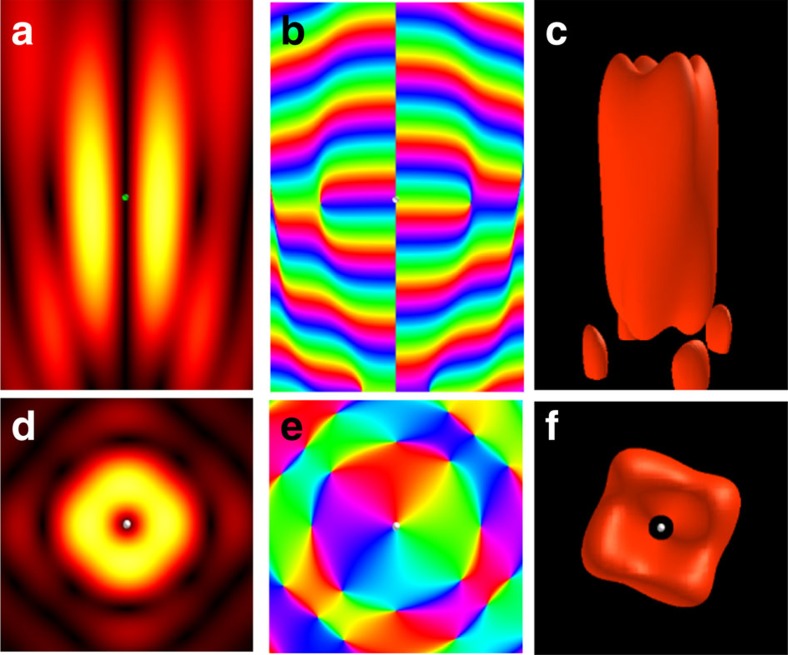
Vortex trap generated with a flat 20 × 20 array 12 cm above the centre. Amplitude field (**a**,**d**), phase field (**b**,**e**) and amplitude isosurfaces of 2 kPa (**c**,**f**).

**Figure 5:**
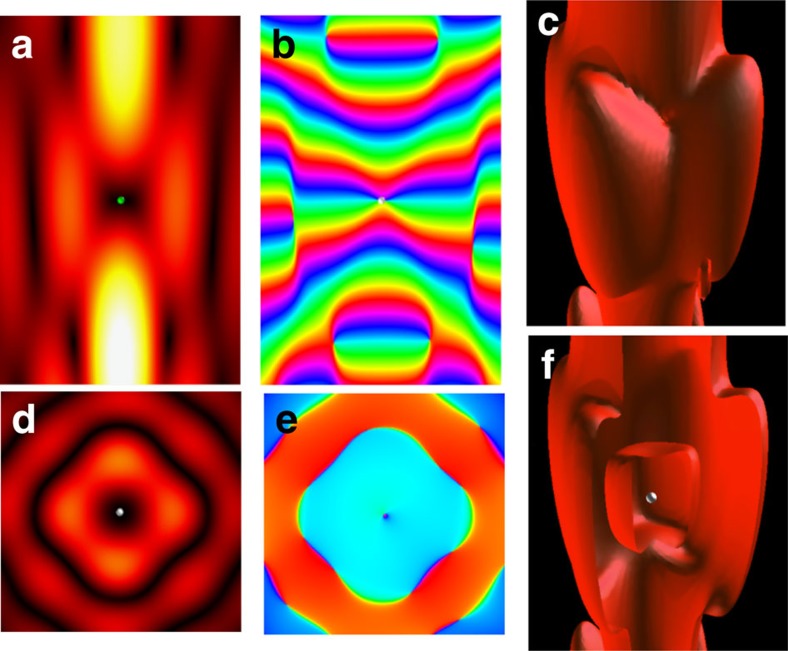
Bottle trap generated with a flat 20 × 20 array 12 cm above the centre. Amplitude field (**a**,**d**) and phase field (**b**,**e**). Amplitude isosurface of 1.3 kPa: full (**c**) and sliced to see the interior (**f**).
