# Supplementary material for: Holographic acoustic elements for manipulation of levitated objects
Source: Nat Commun. 2015 Oct 27;6:8661. doi: 10.1038/ncomms9661 (PMC4627579; doi:10.1038/ncomms9661)
Supplement: Supplementary Information — Supplementary Figures 1-12, Supplementary Tables 1-10 and Supplementary Notes 1-10. [file ncomms9661-s1.pdf]

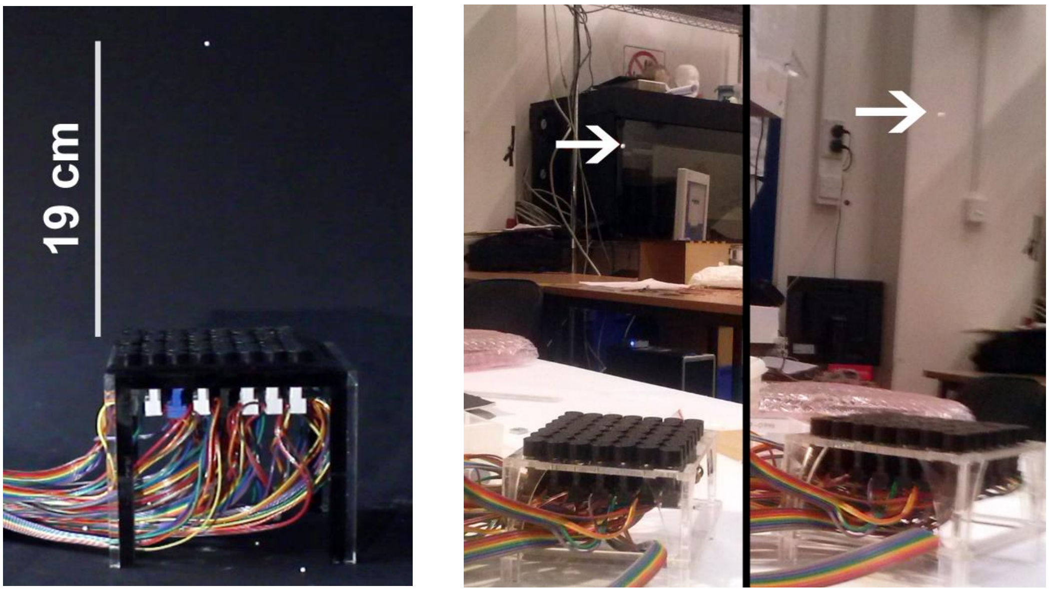

Supplementary Figure 1: Levitation around the focal point. *A bead can levitate around the focal point but it is not a stable trap (See Supplementary Movie 2). The white arrows indicate the position of the levitated particle.*

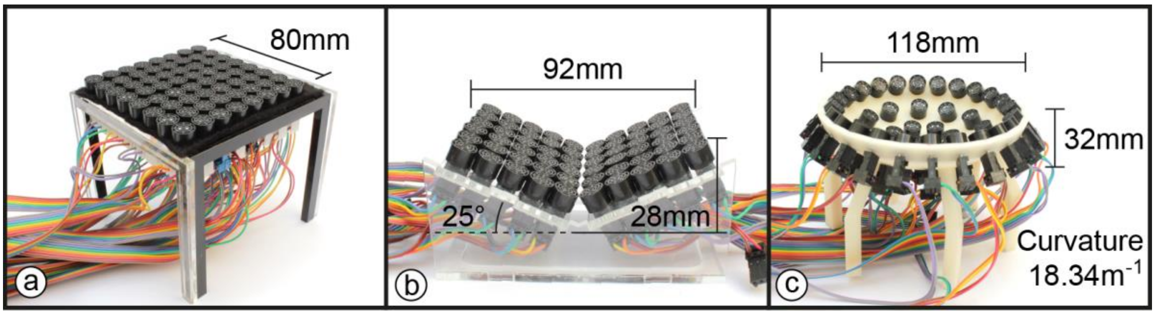

Supplementary Figure 2: One-sided arrangements of transducer*. a) flat: 8x8. b) V-shape: 5x6 per side. c) hemispherical cap with 52 transducers.*

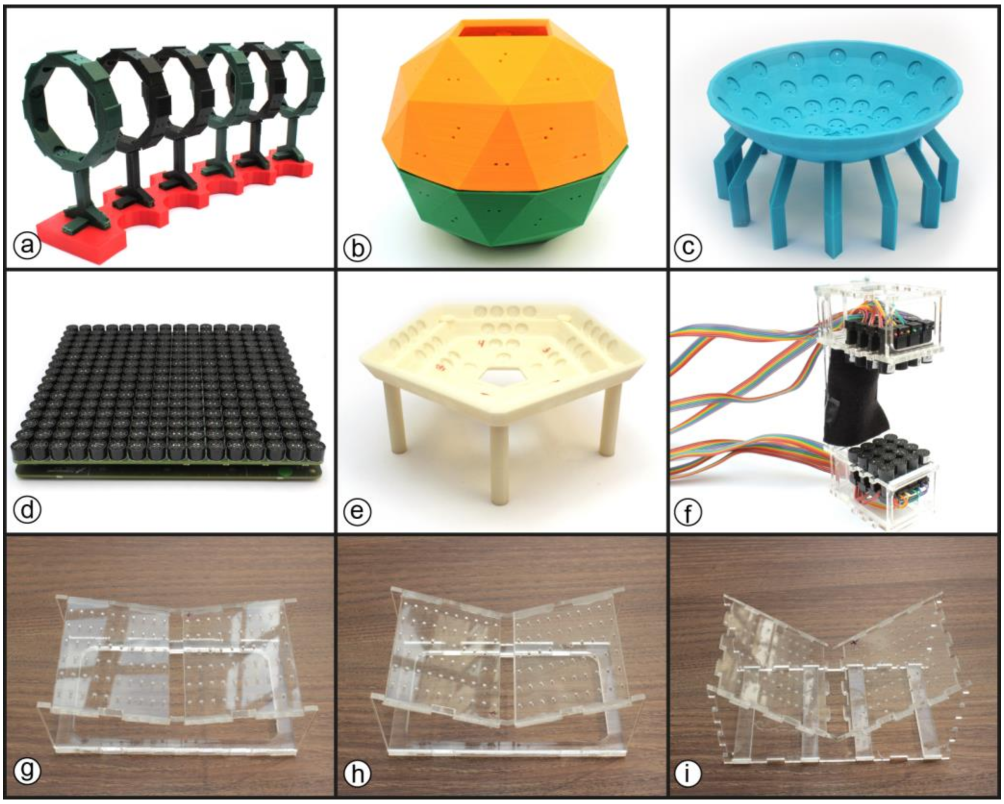

Supplementary Figure 3: Other arrangements used during the experiments. *a) 6 rings forming a curved pipe, 8 transducers per ring. b) two hemispheres of 32 transducers each, they can form a hollow sphere. c) a hemispherical cap with 50 transducers and a different distribution. d) a 16x16 flat array. e) pentagon with 10 transducers per side. f) two opposed arrays separated 62mm with 16 transducers per side. V-shapes with 5x6 transducers forming 8º (g), 18º (h) and 34º(I) with the ground.*

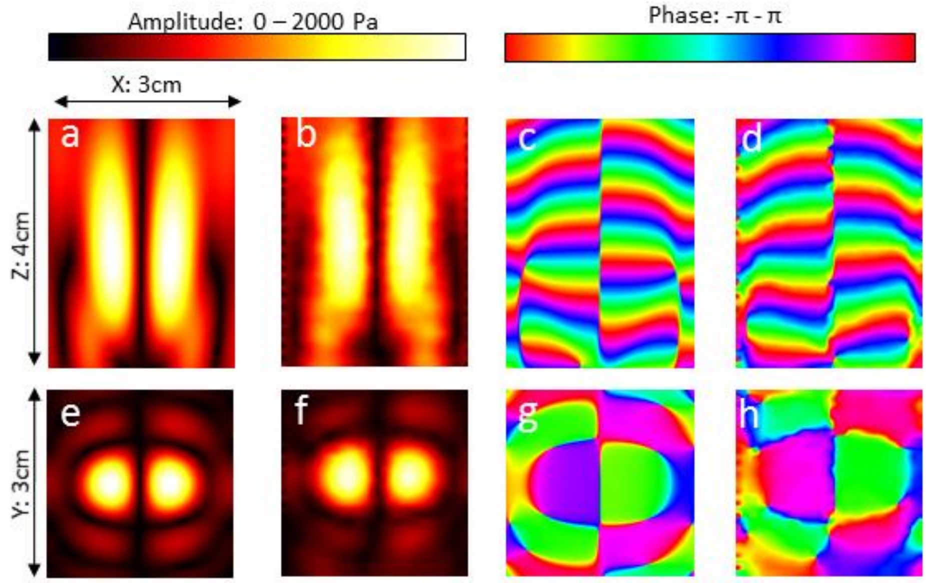

Supplementary Figure 4: Twin Trap generated at the starting position with the flat array. *Simulated (a,e) and experimental (b,f) amplitude. Simulated (c,g) and experimental (d,h) phase.*

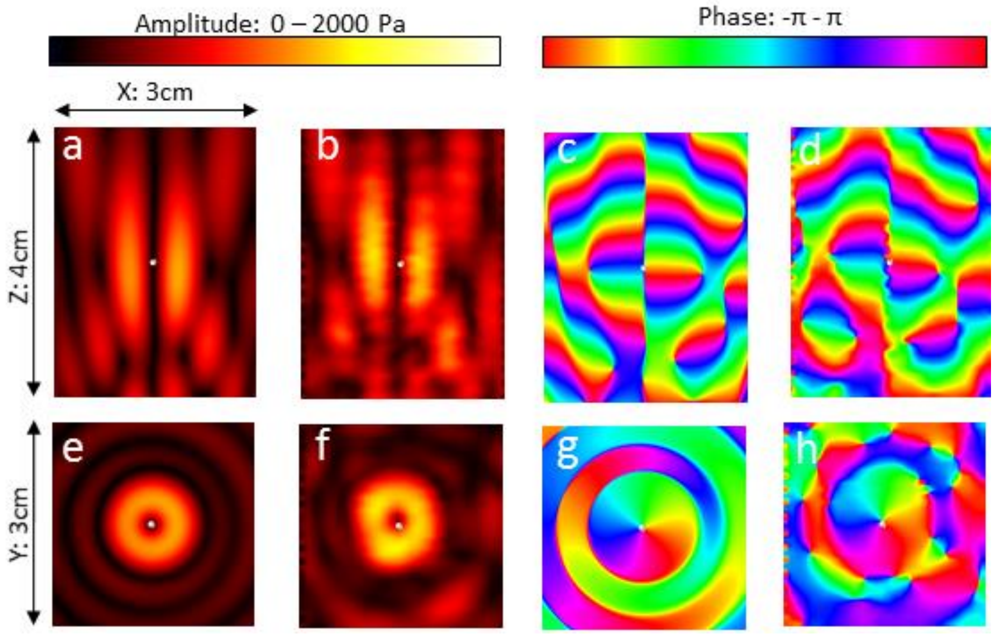

Supplementary Figure 5: Vortex Trap generated at the starting position with the hemispherical cap array. *Simulated (a,e) and experimental (b,f) amplitude. Simulated (c,g) and experimental (d,h) phase.*

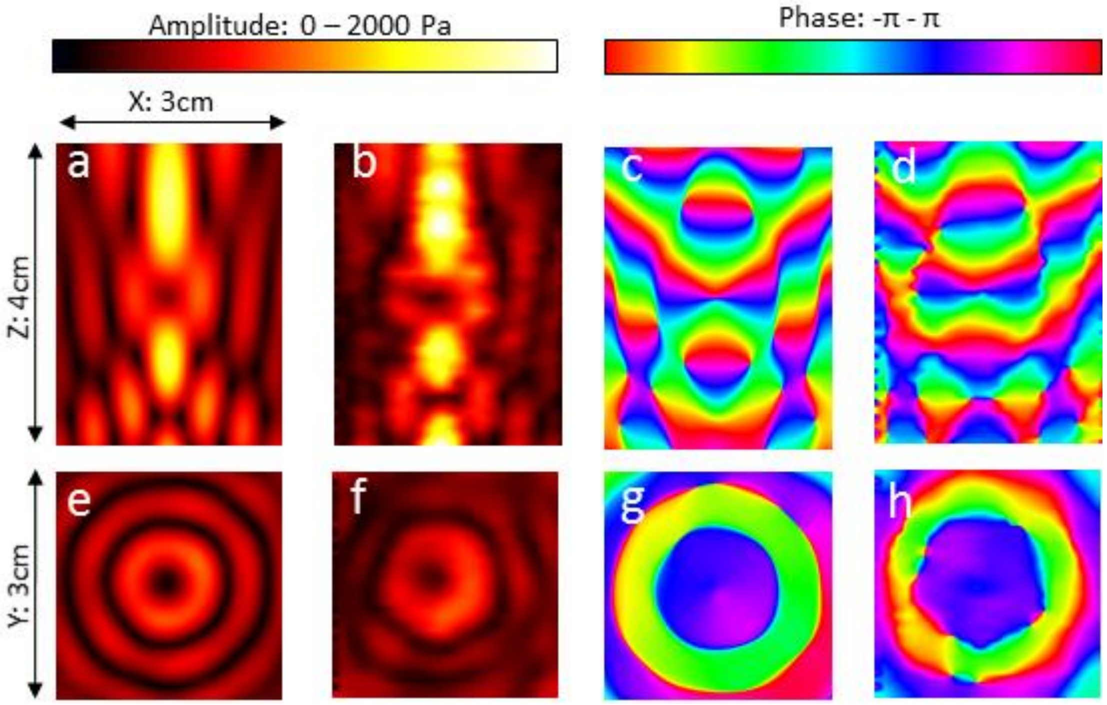

Supplementary Figure 6: Bottle Trap generated at the starting position with the hemispherical cap array. *Simulated (a,e) and experimental (b,f) amplitude. Simulated (c,g) and experimental (d,h) phase.*

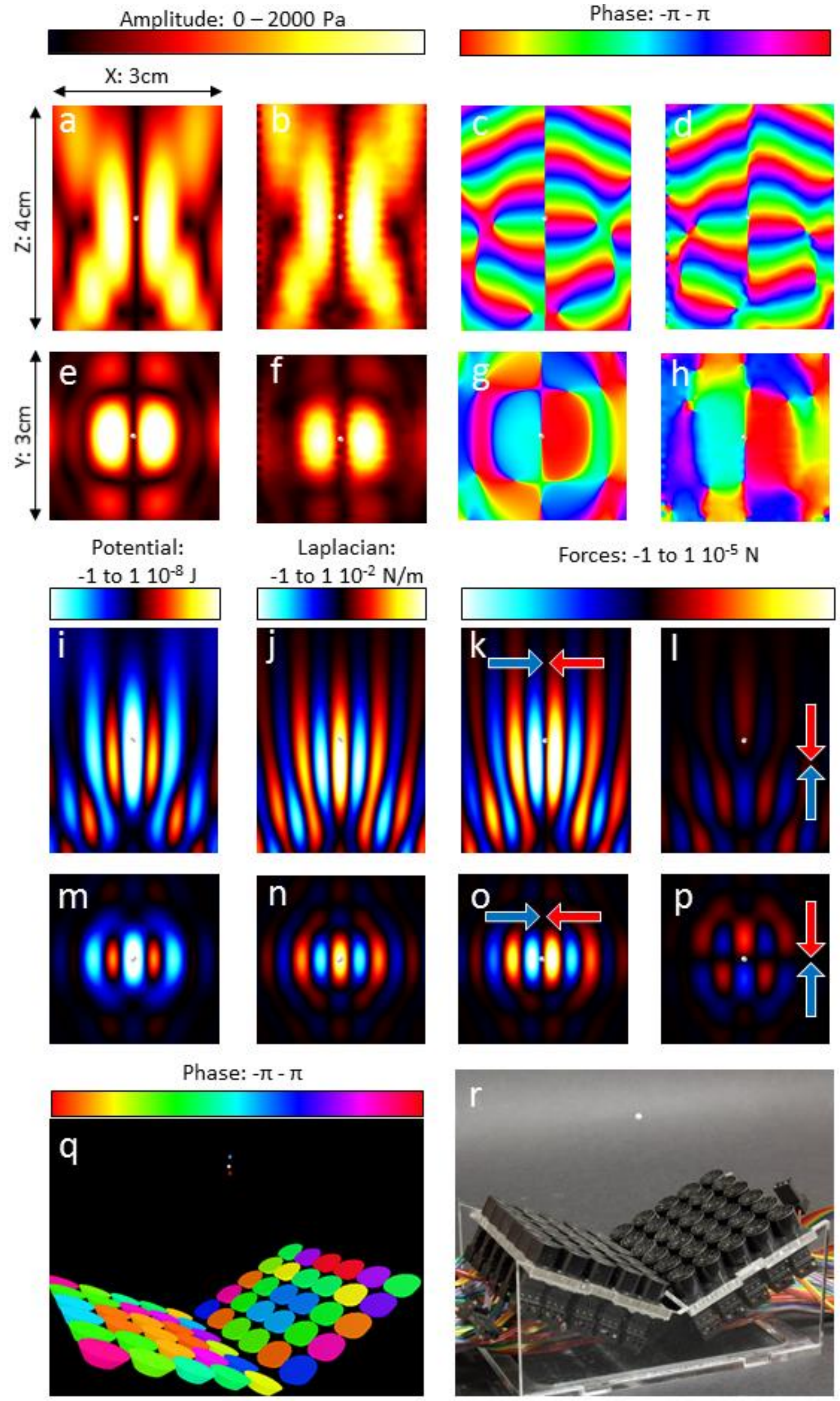

Supplementary Figure 7: Twin Trap generated with the V-shape array 20mm above the transducers. *Simulated (a,e) and experimental (b,f) amplitude. Simulated (c,g) and experimental (d,h) phase. Gor'kov potential (i,m) and Gor'kov Laplacian (j,n). x-component (k,o), z-component (l) and y-component (p) of the forces. Simulated (q) and experimental (r) levitation point. In (q) the colour represents the phase of the transducers. In (k,l,o,p) the red and blue arrows indicate the direction of the forces.*

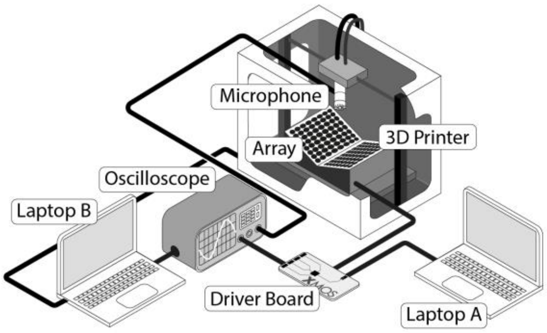

Supplementary Figure 8: Experimental setup for capturing 2D slices of the acoustic field. *Laptop A sends the phase delays to the driver board. The driver board generates a square wave signal (16Vpp and 40KHz with a resolution of 50 phase values per period) and sends it to the transducers; also, a reference signal (0° phase) is sent to the oscilloscope. A calibrated microphone reads the pressure levels created by the transducers and the oscilloscope sends them to Laptop B. Laptop B calculates the amplitude and phase of the microphone readings using the reference signal. Laptop B also controls the 3D stage to move the microphone and scan the field.*

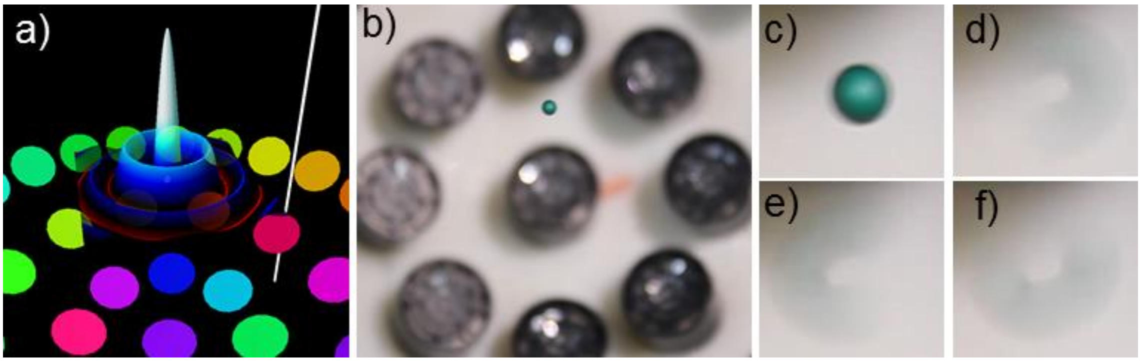

Supplementary Figure 9: Vortex traps potential field. *a) Gor'kov Potential of a Vortex trap. b,c) bead trapped in the central potential well. d,e,f) bead spinning inside the circular potential well before gaining enough speed to be ejected. (See Supplementary Movie 3).*

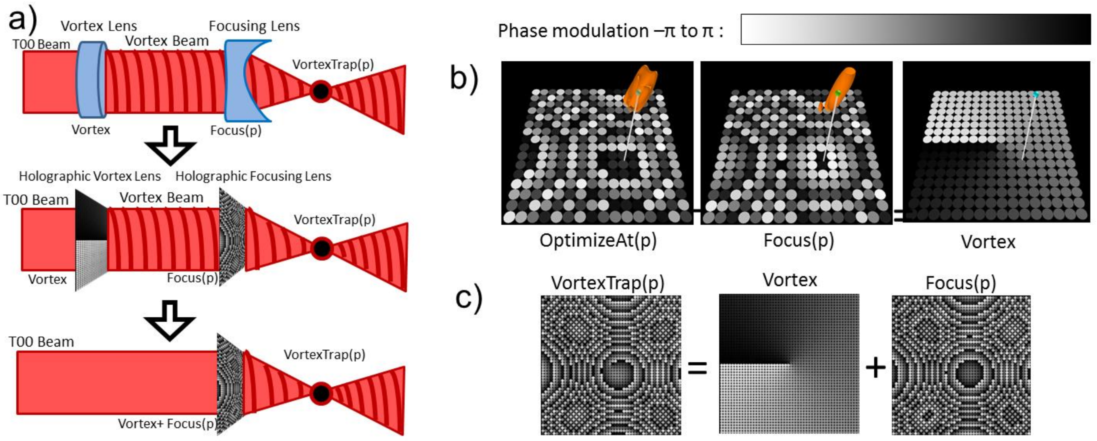

Supplementary Figure 10: Analogy between optic and acoustic Vortex traps. *a) Optical vortex trap created with: physical optical elements (top), holographic optical elements (centre) and a combination of the elements into a single holographic plate. The greyscale represents the phase modulation applied with a Spatial Light Modulator. b) Acoustic equivalent. Here we show that the result of the optimizer for creating a trap minus the phase delays for creating a focal point at the same position is equal to a vortex pattern. The grey scale represents the phase delay of the transducers. c) The holographic plate of a Vortex trap (either acoustic or optic) decomposed into its elements.*

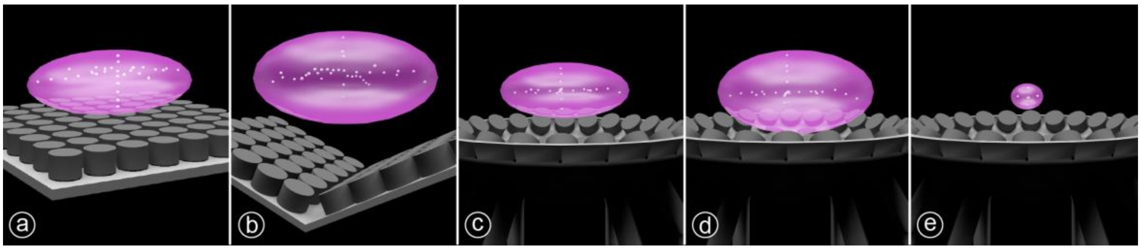

Supplementary Figure 11: Working volume (in pink) of some arrangements and traps. *The white points represent experimental positions that were reachable from the central position. Twin traps in the flat array (a), V-shaped array (b) and hemispherical array (c). Vortex trap (d) and Bottle trap (e) in the hemispherical array.*

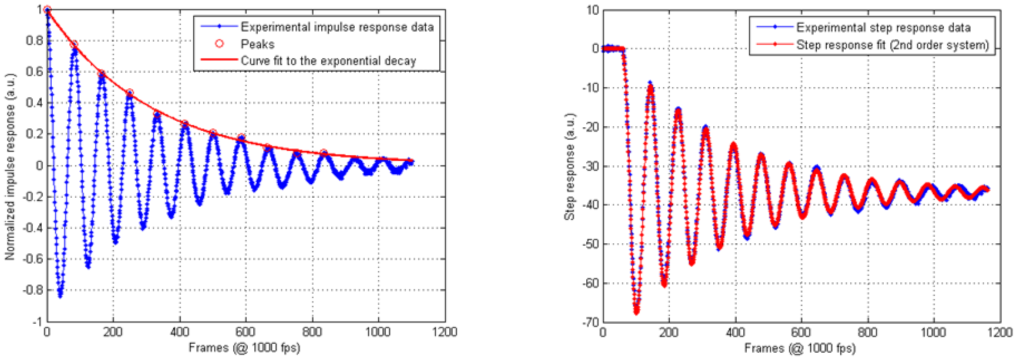

Supplementary Figure 12: Adjusting a damped spring model to the position of the bead. *Left) the damping coefficient. Right) the model in red overlapped on the data in blue.*

		Maximum Linear Speed (cm/s)		
Trap	Arrangement	X	Y	Z
Twin	Flat	1.47	*17.06	12.72
Vortex	Hemisphere	*17.06	*17.06	*17.06
Bottle	Hemisphere	0.54	0.54	0.51
Standing Wave	2 Opposed	*29.00	*29.00	0.51
Twin	V-shape	1.64	*17.06	*25.58
Twin	Hemisphere	2.05	*17.06	*25.58

Supplementary Table 1: Maximum Linear speed for different traps and arrangements. The marked speeds (*) were limited by the electronics of the systems.

Trap	Arrangement	Maximum Angular Speed (RPM)
Twin	Flat	128.88
Twin	Hemisphere	210.13

Supplementary Table 2: Maximum Angular speed for different traps and arrangements.

		Position Error (mm)					
Trap	Arrangement	**X**	SD	**Y**	SD	**Z**	SD
Twin	Flat	**0.11**	0.01	**0.44**	0.02	**0.14**	0.05
Vortex	Hemisphere	**0.07**	0.01	**0.26**	0.01	**0.08**	0.03
Bottle	Hemisphere	**0.06**	0.01	**0.08**	0.01	**0.08**	0.01
Standing Wave	2-Opposed	**0.06**	0.01	**0.05**	0.01	**0.07**	0.01
Twin	V-shape	**0.07**	0.01	**0.10**	0.01	**0.23**	0.03
Twin	Hemisphere	**0.17**	0.02	**0.26**	0.03	**0.28**	0.03

Supplementary Table 3: Repositioning error for translation on different traps and arrangements.

		Angular Error (degrees)	
Trap	Arrangement	**Angle**	SD
Twin	Flat	**2.19**	1.25
Twin	Hemisphere	**4.11**	4.71

Supplementary Table 4: Repositioning error for rotation on different traps and arrangements.

Trap	Arrangement	Linear Spring Constants (mN/m)					
		**X**	SD	**Y**	SD	**Z**	SD
Twin	Flat	**4.68**	0.24	**1.60**	0.08	**0.28**	0.01
Vortex	Hemisphere	**0.26**	0.03	**0.27**	0.04	**0.06**	0.00
Vortex*	Hemisphere	**2.28**	0.29	**2.36**	0.38	**0.49**	0.02
Bottle	Hemisphere	**0.48**	0.01	**0.46**	0.02	**3.54**	0.11
Standing Wave	2 Opposed	**3.14**	0.03	**2.85**	0.05	**14.90**	0.07
Twin	V-shape	**6.11**	0.33	**1.72**	0.03	**0.53**	0.01
Twin	Hemisphere	**7.46**	0.16	**2.09**	0.08	**0.48**	0.01

Supplementary Table 5: Linear Spring Constants split by arrangement, trap and axis. (*) Extrapolation for a particle of the same size as the one used in the rest of the traps.

Trap	Arrangement	Torsional Spring Constant (pN-m/rad)	
		**Angle**	SD
Twin	Flat	**1.56**	0.13
Twin	Hemisphere	**1.82**	0.17

Supplementary Table 6: Torsional Spring Constants split by arrangement, trap and axis.

Trap	Arrangement	Maximum Linear Step (mm)		
		X	Y	Z
Twin	Flat	2.4	3.3	7.5
Vortex	Hemisphere	3	3	7.7
Bottle	Hemisphere	2.6	2.6	2.3
Standing Wave	2 Opposed	7.3	7.1	2.2
Twin	V-shape	2.3	5.3	8.2
Twin	Hemisphere	2.4	3.6	6.5

Supplementary Table 7: Maximum step size that is possible to apply for translation on different traps and arrangements.

Trap	Arrangement	Maximum Angular Step (degrees)
Twin	Flat	55
Twin	Hemisphere	67

Supplementary Table 8: Maximum step size that is possible to apply for rotation on different traps and arrangements.

		Position Model Accuracy (wavelength)					
Trap	Arrangement	**X**	SD	**Y**	SD	**Z**	SD
Twin	Flat	**0.10**	0.18	**0.01**	0.08	**0.28**	0.01
Vortex	Hemisphere	**0.03**	0.01	**0.33**	0.20	**0.18**	0.21
Bottle	Hemisphere	**0.19**	0.16	**0.18**	0.09	**0.28**	0.18
Twin	V-shape	**0.04**	0.03	**0.24**	0.18	**0.26**	0.17
Twin	Hemisphere	**0.01**	0.01	**0.33**	0.17	**0.21**	0.16

Supplementary Table 9: Differences between the predicted and experimental levitation position divided by axis, arrangement and trap. Each entry was extracted from at least 8 separate measurements for the Bottle trap and an average of 30 for rest of traps.

		Rotation Model Accuracy (degrees)	
Trap	Arrangement	**Angle**	SD
Twin	Flat	**5.06**	3.01
Twin	Hemisphere	**9.18**	7.40

Supplementary Table 10: Differences between the predicted and the experimental angles. Each entry was extracted from 72 separate measurements.

Supplementary Note 1

Transducer Arrangements

We built and quantified the performance of three representative single-sided arrangements: an 8x8 flat array, a V-shaped array and a hemispherical cap (Supplementary Figure 2); other arrangements were tested (Supplementary Figure 3). Supplementary Figure 2 (a,b) and 3 (f,g,h,i) were built in 3mm acrylic with a laser cutter (VLS2.30, Universal Laser Systems); whereas Supplementary Figure 2 (c) and 3 (a,b,c,e) were 3D printed (Stratasys FDM 400mc and MakerBot Replicator 2).

The transducers used in all the arrays were 1cm-diameter ultrasound transducers (MA40S4S, Murata Electronics, Japan) with a central frequency of 40kHz, a beam spread angle of ±40° (measured at – 6dB points) and sound pressure levels of 120±3 dB (measured on the axis at a z = 30cm). A custom-made driver board, built with L1-128 100 MHz processors (XMOS, Bristol, UK) and regular MOSFETS, was used to drive the transducers and adjust their phases in real-time. The signal used to drive the transducers was a $16V_{pp}$ square-wave signal of 40 kHz. However, the narrowband nature of the transducers make the output waveform sinusoidal, this has been observed experimentally using a wideband calibrated microphone (Type 4138-A-015, Brüel & Kjær, Nærum, Denmark). The driver boards can synchronously send the signals for all the transducers at 2MHz, resulting in a phase resolution of $\pi/25$; the computer can send phases to the driver board every 5ms. The levitated particles were expanded polystyrene (EPS) spheres ranging from 0.6 to 3.1 mm of diameter and a density of 29.36 kg/m^3 (Custompac Ltd., Castleford, UK).

Supplementary Note 2

Experimental Conditions

A 1.84mm diameter particle of EPS (density 29.36 kg/m^3) was used for all the traps except for the Vortex trap that used a 0.89mm particle since big particles get ejected from the trap. The size of the particles was measured with a Microscope (Leica DM2500) and with a camera (Canon EOS500D) while the particle was levitating. The weight of the particles was measured using a micro-balance (Mettler Toledo MX5). For controlled rotation experiments, an ellipsoidal particle of 2.13mm/2.2mm/1.38mm was used. The starting positions were in the centre of the arrays and: 15mm above the flat array, 10mm above the V-shape and the

hemisphere; and in the middle of the two opposed arrays (Supplementary Figure 2.f). Thereby, the distances from the starting position to the transducers was similar for all the arrangements.

Supplementary Note 3

The Acoustic Field Around the Traps

The acoustic pressure field has a complex value at each point, indicating the amplitude and phase. We present the simulated and experimental acoustic fields around some of the traps generated with different arrangements; namely, a Twin trap in the flat array (Supplementary Figure 4), a Vortex trap (Supplementary Figure 5) and a Bottle trap (Supplementary Figure 6) in the hemispherical array. In Supplementary Figure 7, the force, Gor’kov and Laplacian of the Gor’kov as well as the acoustic pressure field are shown for a Twin trap generated with the V-shape.

2D slices of the acoustic field were measured using a 1/8” Brüel & Kjær calibrated microphone (Type 4138-A-015) attached to a three-dimensional translational stage from a 3D printer (UltiMaker 2). For each slice, measurements were obtained using 1mm steps. The signal from the microphone was band-pass filtered at 40 kHz to remove noise and converted into complex pressure values (amplitude and phase). A detailed overview of the setup is presented in Supplementary Figure 8.

The simulated and experimental fields present good agreement considering the resolution of the experimental slices (1mm due to the size of the microphone) and the difficulty of capturing phase singularities.

Supplementary Note 4

Vortex Spin

Vortex traps transfer angular momentum to the levitating particles. Using the high speed camera the spinning speed of the particle was determined to be 4114±144 RPM. The particle was marked with a black dot and the frames between every full rotation counted. 100 full rotations were analysed. When particles bigger than 0.9mm diameter were levitated in the vortex trap, they were ejected from the trap (Supplementary Figure 9). Vortex traps have a powerful

potential well at the levitation point but it is narrow and surrounded by a circular well. Therefore, big particles move out of the point trap and fall into the circular trap. Once there, the particles levitate in circles increasing in speed until being ejected.

Supplementary Note 5

Equivalence between Optical and Acoustic Vortex Traps

In Supplementary Figure 10 it is shown that the holographic elements used for both optical and acoustic Vortex traps are the same. Furthermore, acoustic vortex traps emerge as the solution of our optimization method suggesting that also optical Vortex traps may be an optimal mechanism for trapping.

Supplementary Note 6

Working Volume

The working volume (WV) is the space within which controlled and stable manipulation is possible. Each of the arrangements and trap types has an associated WV due to the combined effect of distance from the transducers, directivity and the characteristics of the traps. In Supplementary Figure 11, we illustrate the WV of some combinations of traps and arrangements.

To experimentally obtain the working volume, the particle was first located at the starting position of the array. Then, it was moved along the primary axes until it dropped out of the trap. Some intermediate positions were tested to get a more detailed working volume.

To determine the 3D position of the levitated particles, two cameras (Canon EOS500D) were placed orthogonally pointing towards the array. The intrinsic parameters of the cameras (focal length, image sensor format, principal point and up to 5th order lens distortion) were calibrated using OpenCV and a checker pattern. The particles and the transducers were manually marked in each image. The positions of the transducers were known and thus the position and orientation of the cameras (extrinsic parameters) could be extracted. Each 2D position of a particle in an image represents a 3D ray emerging from the camera and passing through the particle. Therefore, the closest point between the two rays emerging from the cameras represent the 3D position of the bead. An absolute error of less than 0.1mm was determined.

Supplementary Note 7

Maximum Speed and Step Size

This experiment determines the maximum linear and angular speed of levitated particles by some of the different traps and arrangements (Supplementary Tables 1 and 2). Additionally, the maximum amount of movement that it is possible to apply in one step without the particle falling was measured (Supplementary Tables 7 and 8).

For the maximum speed, the particle was moved back and forth along the tested dimension in a linear path of 10mm, increasing the speed every 10 successful completions of the path. The electronics supported updates every 5ms and 0.1mm steps were used; whenever the maximum update rate was reached, the step size was increased. The speeds marked with (*) were limited by the electronics and higher update rates would enable higher speeds. For the rotation, the speed was increased every 10 successfully complete rotations. For the maximum steps, the step size was considered achievable when it was possible to repeat it 10 times.

Supplementary Note 8

Repositioning Error

This experiment measures the repositioning error. The particles are levitated at the starting position and translated 10mm (using 1mm steps) following the pattern: left, forward, right, up, backward, left, forward, right, backward and down; the object was tracked at each of these 11 positions and the procedure was repeated 4 times. For the rotation measurements, the particle started at 0 degrees and completely rotated 4 times with 20 degrees steps. The deviation between the positions is shown per axis as well as the angular repositioning error in Supplementary Table 3 and 4.

Supplementary Note 9

Trapping Forces: Spring Constants

The trapping strength of different arrangements and traps was measured in this experiment. A damped spring model was fitted to the position of the particle over time while it was moved from one position to another (Supplementary Figure 12). The spring constant was used as a measure of the stiffness strength of the trap.

The particles were displaced 2mm for linear spring constants and 45 degrees for torsional constants; and returned to their starting position while recorded with a high speed camera (Photron SA-1) at up to 3000FPS. The procedure was repeated 5 times for each constant. The linear and torsional spring constants are presented in Supplementary Table 5 and 6.

The experimental spring constants are consistent with the experimental observation that a particle was levitating around 2.5mm below the trap centre (flat array and conditions from Supplementary Note 2), where the spring force was equal to mass multiplied by gravity and around 0.7 μN. Spring constants also agree with the simulated forces such as the ones in Figure S4.

Supplementary Note 10

Model Accuracy

This experiment compared the predicted levitation positions and the real levitation position. In Supplementary Table 9, the deviation from the predicted and real positions are shown per axis; and in Supplementary Table 10 the angle deviation. The positions tested were the same from Supplementary Note 6 (Working Volume). For the rotation measurements, the particle started at 0 degrees and completely rotated 4 times with 20 degrees steps.

Disagreements between the levitation positions could be due to imperfections in the construction of the arrangements and socketing of the transducers on the base. Additionally, the Piston model is a simple way of predicting the far-field that was used because modelling accuracy was not the focus of the paper, more accurate and sophisticated models can be used.
